# TREM2 in the pathogenesis of AD: a lipid metabolism regulator and potential metabolic therapeutic target

**DOI:** 10.1186/s13024-022-00542-y

**Published:** 2022-06-03

**Authors:** Rui-Yang Li, Qi Qin, Han-Chen Yang, Ying-Ying Wang, Ying-Xin Mi, Yun-Si Yin, Meng Wang, Chao-Ji Yu, Yi Tang

**Affiliations:** 1 Innovation Center for Neurological Disorders, Department of Neurology, Xuanwu Hospital, Capital Medical University, National Center for Neurological Disorders, Beijing, China; 2 State Key Laboratory of Stem Cell and Reproductive Biology, Institute of Zoology, Chinese Academy of Sciences, Beijing, China

**Keywords:** Alzheimer’s disease, Central nervous system, Lipid metabolism, Peripheral system, Therapeutic target, TREM2

## Abstract

Triggering receptor expressed on myeloid cells 2 (TREM2) is a single-pass transmembrane immune receptor that is mainly expressed on microglia in the brain and macrophages in the periphery. Recent studies have identified TREM2 as a risk factor for Alzheimer’s disease (AD). Increasing evidence has shown that TREM2 can affect lipid metabolism both in the central nervous system (CNS) and in the periphery. In the CNS, TREM2 affects the metabolism of cholesterol, myelin, and phospholipids and promotes the transition of microglia into a disease-associated phenotype. In the periphery, TREM2 influences lipid metabolism by regulating the onset and progression of obesity and its complications, such as hypercholesterolemia, atherosclerosis, and nonalcoholic fatty liver disease. All these altered lipid metabolism processes could influence the pathogenesis of AD through several means, including affecting inflammation, insulin resistance, and AD pathologies. Herein, we will discuss a potential pathway that TREM2 mediates lipid metabolism to influence the pathogenesis of AD in both the CNS and periphery. Moreover, we discuss the possibility that TREM2 may be a key factor that links central and peripheral lipid metabolism under disease conditions, including AD. This link may be due to impacts on the integrity of the blood–brain barrier, and we introduce potential pathways by which TREM2 affects the blood–brain barrier. Moreover, we discuss the role of lipids in TREM2-associated treatments for AD. We propose some potential therapies targeting TREM2 and discuss the prospect and limitations of these therapies.

## Background

With the general increase in the lifespan of the population worldwide, Alzheimer’s disease (AD) is rapidly becoming a major disease burden and socioeconomic challenge [1]. AD is a progressive neurodegenerative disease characterized by latent memory and cognitive loss [2]. According to the World Alzheimer Report of 2019, AD is the most common form of dementia in older adults, with about 50 million people worldwide diagnosed with AD or AD-related dementia. At present, AD pathogenesis is a research hotspot. Various factors have been shown to affect AD pathogenesis, ranging from neuroinflammation and brain pathologies to molecules, such as some kinds of proteins and RNA [3–6].

Recently, an increasing number of researchers have considered triggering receptor expressed on myeloid cells 2 (TREM2) to be a factor that influences AD pathogenesis [7]. TREM2 is a single-pass transmembrane immune receptor that is mainly expressed on microglia in the brain [8, 9] and macrophages in the periphery [10]. The signaling pathways of TREM2 are complex. In general, TREM2 binds to the adaptor proteins DNAX activation protein 10 (DAP10) and DAP12 through oppositely charged residues in their transmembrane domains [11]. As reviewed by Deczkowska et al., DAP10 and DAP12 are phosphorylated and mediate intracellular signal transduction mechanisms upon TREM2-ligand interaction. DAP10 mediates signal transduction by activating phosphatidylinositol 3-kinase, while DAP12 promotes the activation of splenic tyrosine kinase [11]. TREM2 binds to DAP10 or DAP12 and can form TREM2-DAP12-DAP10 heterodimers, which can then mediate downstream signaling [12, 13]. Although the function of TREM2 is not completely understood, TREM2 has been found to regulate inflammatory signaling [14] and microglial metabolism [9] and could promote microglial phagocytosis [8, 15, 16], activation [8, 17], survival [8, 9] and proliferation [15]. Thus, it is important for normal immune function and cell viability in the brain. In addition, TREM2 is a confirmed genetic risk factor for AD [18], and most studies evaluating the association between TREM2 and AD focused on tau pathology and amyloid-β peptide (Aβ) pathology. Other than the widely studied effects of TREM2 function in the context of either Aβ [8, 19] or tau pathologies [20], a recent study found that TREM2 acts at a critical intersection of Aβ and tau pathologies, affecting AD pathogenesis by limiting Aβ plaque-mediated tau pathologies [21].

In addition to these pathologic changes, lipids also influence the pathogenesis of AD. Interestingly, TREM2 was found to have a vital role in both central and peripheral lipid metabolism. Recent researches have shown that TREM2 can bind to lipid-associated ligands, such as phospholipids [8], high-density lipoproteins, low-density lipoproteins (LDL) [22], lipids contained in apoptotic neurons [23], and apolipoprotein E (ApoE), which is an important lipid transporter in the CNS [22, 24, 25]. These associations indicate a certain relationship between TREM2 and lipids. In the CNS, TREM2 affects cholesterol and myelin metabolism in the brain [26, 27], binds to phospholipids, and affects the metabolism of some types of phospholipids [28, 29]. In addition, TREM2 promotes the microglial transition to disease-associated microglia (DAM) through several lipid-related pathways [30], which in turn enhances lipid metabolism in the CNS [31]. These alterations might affect AD by influencing cerebrovascular function, the brain immune response or AD pathologies. In the periphery, TREM2 is associated with the occurrence and progression of obesity and its complications, such as hypercholesterolemia, atherosclerosis, and nonalcoholic fatty liver disease (NAFLD). These diseases are considered metabolic comorbidities of AD [32–35]. Notably, these diseases are characterized by a series of lipid metabolism disorders, and their development further disrupts the homeostasis of peripheral lipid metabolism [36–41]. The disorder of peripheral lipid metabolism can induce peripheral inflammation and IR, which may lead to central inflammation and IR and further promote the pathogenesis of AD. These findings suggest that the mechanisms by which TREM2 affects obesity and its complications might be potential mechanisms by which TREM2 affects peripheral lipid metabolism and AD pathogenesis.

In this review, we discuss the possibility that lipid metabolism is a novel pathway by which TREM2 affects AD pathogenesis both in the CNS and the periphery. We also discuss the possibility that TREM2 is a key factor that links central and peripheral lipid metabolism under disease conditions, including AD, likely by influencing the integrity of the blood–brain barrier (BBB). Moreover, we discuss the role of lipids in TREM2-associated treatments for AD. We propose some potential therapies targeting TREM2 and discuss the prospect and limitations of these therapies.

### TREM2 regulates lipid metabolism in the CNS

For the past few years, studies on TREM2 have mainly focused on its role in the CNS. TREM2 has a highly characteristic expression in the CNS. At the anatomical level, TREM2 is highly expressed in the corpus callosum and basal ganglia [42], while at the cellular level, TREM2 is selectively expressed in microglia [26]. TREM2 was identified as a lipid receptor and can regulate cholesterol and phospholipid metabolism in the CNS [26, 43]. In addition, TREM2 promotes the microglial transition to DAM, which interacts with lipid metabolism in the brain [31] and influences AD pathogenesis [44, 45]. Interestingly, emerging evidence has shown that TREM2 binds to ApoE [22, 46, 47], which is an important lipid transporter in the CNS. Human ApoE protein exists in three major isoforms, ApoE2, ApoE3, and ApoE4, which are encoded by the ε2, ε3, and ε4 alleles of the APOE gene, respectively. All three isoforms can bind to TREM2. APOE ε4 is the largest genetic risk factor for late-onset AD, while APOE ε2 is protective for AD [25, 46]. The mouse ApoE protein is most similar to that of human ApoE3, which is the most common form of ApoE protein [48]. ApoE mediates the endocytosis and efflux of lipids and cholesterol [49, 50] and is indispensable in the transition to DAM [51]. Thus, it is possible that the effect of TREM2 on these lipid metabolism-associated pathways is related to ApoE. Besides, in addition to discussing the TREM2-affected lipid metabolism pathways in the CNS and their relationship with ApoE, we also discuss the possibility that the disordered TREM2-affected lipid metabolism in the CNS contributes to the pathogenesis of AD.

#### TREM2 regulates brain cholesterol and myelin metabolism

Increasing evidence indicates that TREM2 function is associated with lipid metabolism in microglia [15]. However, previous researches mainly focused on lipids, which are potential ligands of TREM2, in the form of either cell surface-exposed signals or lipoprotein particles [19, 46, 47, 52].

**Fig. 1:**
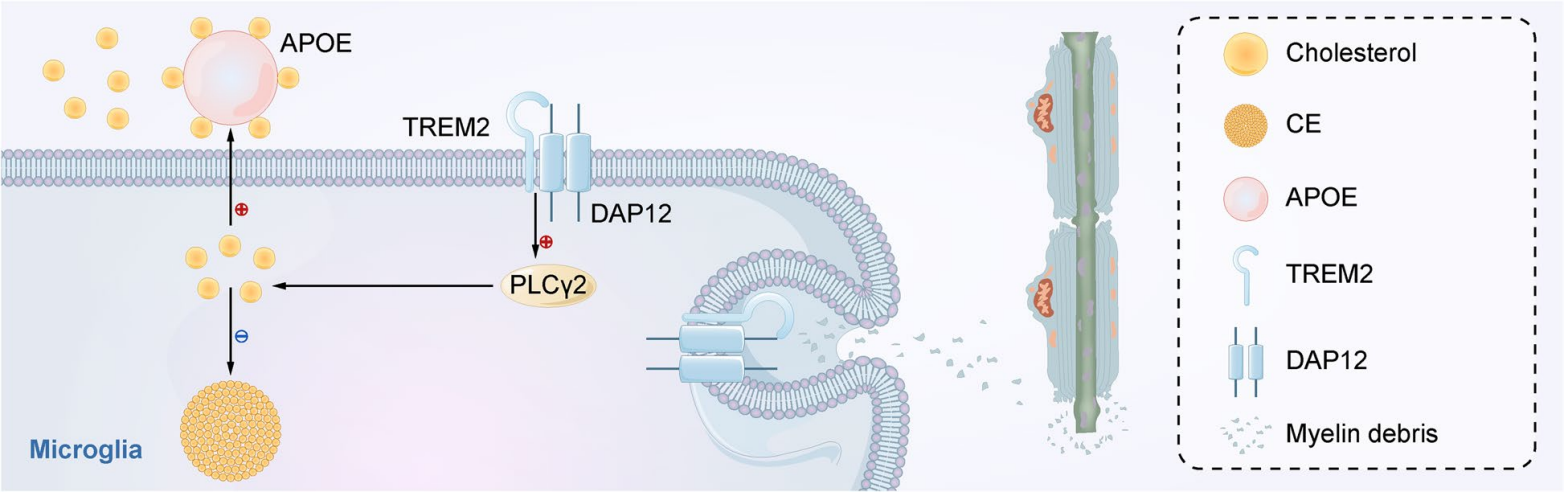
TREM2 regulates microglial cholesterol metabolism and participates in the response to myelin damage. The activation of the TREM2-DAP12 signaling pathway may promote microglial cholesterol efflux and reduces intracellular cholesterol to be stored as CE, possibly by activating PLCγ2. The effluent cholesterol might be carried by APOE-containing lipoproteins. Besides, TREM2 mediates the microglial response to myelin damage, leading to increased phagocytosis of myelin debris and thus may promote remyelination.

Recently, exciting research has established a crucial role of TREM2 signaling in controlling microglial cholesterol metabolism. In a cuprizone (CPZ)-treated model, which is used to simulate demyelination and the effect of demyelination-induced lipid overload, Nugent et al. observed that microglia in Trem2-/- mice have a more than tenfold increase in cholesterol esters (CEs) and oxidized CEs compared with Trem2 + / + mice [26]. To elucidate the mechanisms of CE accumulation, these researchers inhibited the endoplasmic reticulum (ER) enzyme acetyl-CoA acetyltransferase 1 to reduce the synthesis of CEs from free cholesterol and upregulated the cholesterol transporters ABCA1 and ABCG1. Both interventions reduced the accumulation of CE in Trem2-/- mouse models, suggesting that Trem2 deficiency is associated with microglial cholesterol efflux defects or intracellular cholesterol to be stored as CE [26]. Lipidomic analysis of human induced pluripotent stem cell (iPSC)-derived microglia (iMG) further confirmed and extended the lipid regulatory role of TREM2. PLCG2 is an AD-linked gene encoding the intracellular enzyme PLCγ2 which cleaves the membrane phospholipid phosphatidylinositol-4,5-bisphosphate (PIP2) to inositol-1,4,5-trisphosphate (IP3) and diacylglycerol (DAG) [53, 54]. By using lipidomic analysis of cell extracts, Andreone et al. measured the levels of 100 lipids in TREM2 KO iMG and phospholipase C γ2 (PLCG2) KO iMG after exposure to myelin [43]. These cells showed similar increases in several types of lipids, including CEs, free cholesterol, ceramides, sulfatides, phospholipids, triacylglycerols and DAGs, compared to wild type (WT) cells exposed to myelin. Many of these lipids, including CE and free cholesterol, were verified in other independent TREM2 KO iMG clones and PLCG2 KO iMG clones generated by different strategies [43]. Considering that intracellular cholesterol is usually stored as CE [55] and that PLCγ2 signals downstream of the TREM2-DAP12 [43], TREM2 might regulate human microglial cholesterol transport in a PLCγ2-dependent manner (Fig. 1).

Brain cholesterol is produced locally because the BBB prevents cholesterol-rich lipoproteins from entering the CNS. Eighty percent of free cholesterol in the brain is present in the myelin sheath, which is formed by oligodendrocytes to insulate axons. Thus, myelin is a vital and sensitive marker of cholesterol metabolism in the brain [56]. It has been shown that TREM2 participates in the microglial response to myelin damage and thus affects remyelination [27, 57] (Fig. 1). A recent study found that Trem2–/– microglia are unable to amplify transcripts related to activation, lipid catabolism and phagocytosis in response to myelin damage, leading to impaired myelin debris removal, fewer oligodendrocytes, axonal dystrophy, and persistent demyelination. These results, especially the altered clearance of myelin debris (a process required for proper remyelination [27]) and the decrease in oligodendrocytes, could in turn affect the remyelination process [15].

Recently, several other studies have also demonstrated a strong association between TREM2 and myelin metabolism. Piccio’s research showed that intraperitoneal administration of a TREM2-activating monoclonal antibody increased the clearance of myelin fragments, promoting remyelination and the preservation of axonal integrity [58]. Moreover, a recent study identified TREM2 + microglia, a specific microglial subtype with upregulated expression of Trem2, after the development of axonal CNS lesions. This special subtype of microglia was found to be more effective in myelin phagocytosis and thus may promote remyelination as well [59]. Collectively, these studies suggest a protective function of TREM2 in the microglial response to myelin damage. It could possibly influence microglial transcriptional programs to enhance lipid metabolism and myelin debris clearance, thereby promoting remyelination.

Interestingly, several studies suggested that APOE might be involved in this process. In mouse models of demyelination, the expression of Apoe was found to be upregulated in Trem2 + / + and Trem2 ± microglia compared with Trem2-/- microglia [26]. This result suggests that Trem2 might regulate the expression of ApoE during myelin damage. In addition, human studies have found that TREM2 regulates the uptake of ApoE and LDL [22] in microglia. Combined with the observation that Trem2-/- and Apoe-/- mice fed CPZ showed similar accumulation of CE in the brain, the defect of microglial cholesterol efflux upon TREM2 loss-of-function may be caused by reduced efflux of cholesterol onto ApoE-containing lipoproteins [26], which may therefore inhibit intracellular Aβ degradation [60].

#### TREM2 signaling could be induced by binding to phospholipids, and TREM2 regulates phospholipid metabolism

Phospholipids are considered an important biomarker of AD [28]. Although the myelin sheath contains certain types of phospholipids, we will still discuss its relationship with TREM2 separately due to the diversity of phospholipids. The majority of studies consider phospholipids to be ligands for TREM2. Previous studies found that TREM2 binds to damage-associated phospholipids, including phosphatidylserine (PS), and can act as a scavenger receptor for apoptotic cells that may occur during neuronal injury [23].

In the brains of mice with AD, large amounts of PS and phosphatidyl ethanolamine (PE) are exposed to synaptosomes. This may induce TREM2-mediated intracellular signaling [29], thus promoting microglial functions such as phagocytosis, proliferation, survival, and synaptic pruning [8, 15, 61], which is consistent with the findings in human TREM2 reporter cells phosphatidylinositol (PI) and phosphatidylcholine (PC) [8]. In addition, recent lipidomics found that major phospholipid classes, such as PS, PE, phosphatidylcholine (PC) and phosphatidylinositol (PI), are significantly reduced in ApoE4 lipoproteins compared to ApoE3 lipoproteins [62]. It would be interesting to determine whether these changes account for the greater influence of Trem2 deficiency on the microglial uptake of ApoE4-Aβ complexes compared to ApoE3-Aβ [62].

In addition, recent studies have investigated the relationship between TREM2 and phospholipid metabolism. Lipidomics showed increased levels of phospholipids such as PS, PE, PI and PC in TREM2 KO microglia after exposure to myelin [26, 43]. However, none of these studies illuminated the specific mechanisms by which TREM2 influences the metabolism of these phospholipids. Other researches have mainly focused on the metabolism of PI and its derivatives, such as PIP2. A protein–protein interaction network analysis suggested that TREM2 interferes with the metabolism of PI by interacting with downstream of kinase 3 (DOK3) [28], which is an adaptor protein that has a close physical interaction with TREM2 and DAP12 [28, 63]. Nevertheless, additional mechanistic and functional studies are needed to further validate and explore this pathway.

PLCγ2 is an intracellular enzyme that cleaves membrane PIP2 to DAG and IP3 [43]. It signals downstream of TREM2-DAP12, which supports the finding that stimulating the TREM2-DAP12 signaling pathway promotes the hydrolyzation of PIP2 into IP3 at the plasma membrane [64]. Therefore, it is possible that when AD-risk mutations of TREM2 impair the activation of DAP12, the levels of PIP2 in the plasma membrane will increase as a result of the inhibition of PLCγ2 activities [64]. This will in turn suppress the protective function of TREM2 against AD [65].

In addition, PIP2 has been confirmed to mediate ApoE-associated AD pathogenesis [66]. Thus, it is essential for future studies to explore whether the influence of TREM2 on PIP2 metabolism is associated with or even dependent on ApoE.

#### TREM2 promotes the microglial transition to DAM

Disease-associated microglia (DAM) is a specific microglial subtype observed in aging and neurodegenerative conditions. They have become a hot topic in recent years due to their intimate connection with AD. Previous studies have shown that DAMs have a unique signature that shows enhanced microglial phagocytosis and can restrict Aβ plaque growth [30, 67]. These findings suggest that DAM is a potential protective subtype of microglia in AD.

By using Trem2-/- 5XFAD mice, Keren et al. identified two successive stages during the activation of DAM. The first stage is Trem2-independent and involves the activation of genes such as ApoE and tyrosine protein tyrosine kinase-binding protein (Tyrobp, which encodes DAP12). The second step is Trem2-dependent; this step is characterized by the induction of phagocytic pathways and lipid metabolism (e.g., CD9, Lpl, and Cst7) [30]. Thus, it is possible that there is an association between Trem2 and lipid metabolism during the activation of DAM. In addition, not only the Trem2-dependent DAM activation process but also DAM itself shows a close relationship with Trem2 and lipids. Several studies have detected the upregulation of Trem2 [18, 68] and the enhancement of lipid metabolism pathways [30] in DAM through the examination of gene signatures. These findings indicate that the effect of Trem2 on DAM transition may further influence lipid metabolism in the brain.

**Fig. 2:**
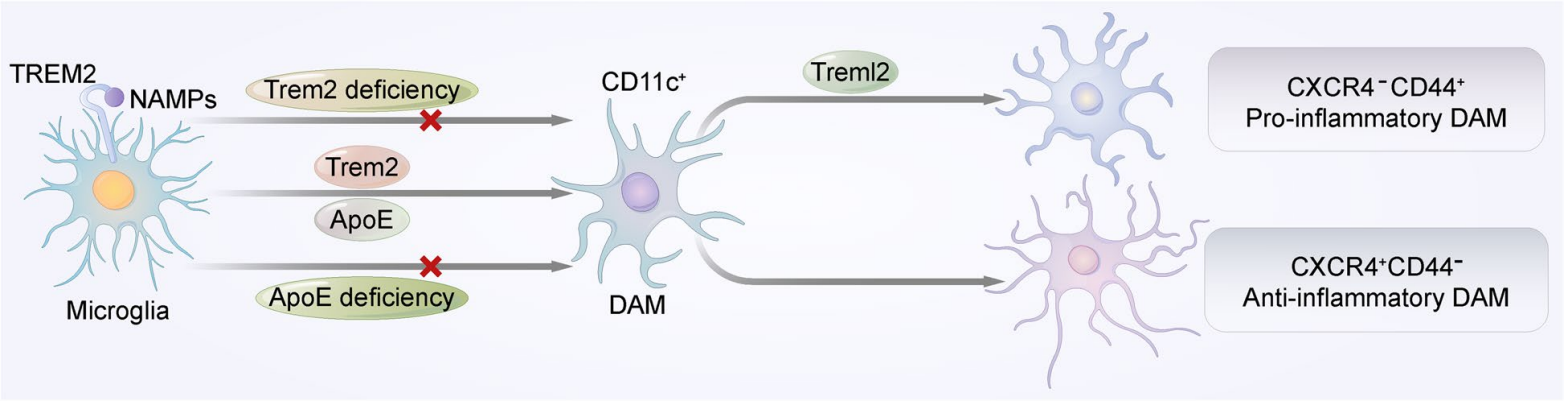
TREM2 promotes the microglial transition to DAM. TREM2 binds to NAMPs and then triggers the microglial transition to DAM (CD11c +). In mouse models, microglia deficient in either Trem2 or ApoE failed to convert to DAM. Pro-inflammatory and anti-inflammatory phenotypes have been identified in DAM. Pro-inflammatory DAM was characterized by surface marker CD44, while anti-inflammatory DAM expressed surface marker CXCR4. Both of these phenotypes occur downstream of Trem2, while the presence of pro-inflammatory DAM also requires the expression of Treml2

To study the specific mechanisms by which Trem2 influences DAM activation, Deczkowska and his colleagues proposed the concept of neurodegeneration-associated molecular patterns (NAMPs). NAMPs are damage-related signals present in the CNS, such as danger molecules present on myelin debris, lipid degradation products and the apoptotic bodies of dying neural cells. Many of these NAMPs are lipids that can be recognized by TREM2, and can then trigger the microglial transition to DAM [8, 15, 69] (Fig. 2). In addition, according to these findings, both phospholipids and myelin are NAMPs. This is aligned with the recent observation that during demyelination, Trem2 expression in microglia upregulates several lipid metabolism-associated DAM gene signatures [30], suggesting that TREM2 might link phospholipids, myelin metabolism and the transition to DAM.

Interestingly, recent studies have shown that the influence of TREM2 on DAM may be associated with APOE. In genetic knockout mouse models, Apoe and Trem2 were found to exert similar effects on DAM, and microglia deficient in either Trem2 or Apoe failed to convert to DAM [51] (Fig. 2). The finding that the inhibition of Trem2 suppressed Apoe expression in APP-PS1 microglia suggests that there might be a TREM2-APOE signaling pathway that is involved in the DAM transition during AD [51]. However, additional mechanistic and functional studies are needed to confirm and explore this signaling pathway, as a study in 5XFAD mice found that Apoe is activated in the Trem2-independent stage in the DAM transition [30].

Notably, recent studies found that DAM consists of different subtypes [67, 70]. Weighted correlation network analysis identified the pro-inflammatory and anti-inflammatory phenotypes of DAM (Fig. 2). The anti-inflammatory phenotype also showed enhanced phagocytosis, lipid metabolism and cholesterol efflux, which was more aligned with the previously acknowledged protective function of DAM. However, although pro-inflammatory DAM seems to be detrimental to AD, both of these phenotypes occur downstream of the immune checkpoint of Trem2 [67]. The presence of pro-inflammatory DAM also requires the expression of Treml2, an AD risk gene with an opposite effect to Trem2 in microglia [71]. These findings indicate that the original role of Trem2 might be to promote the transition to anti-inflammatory DAM. Additionally, the emergence of these two different phenotypes can be regulated by drugs. Thus, they may exert therapeutic effects on a pathological level, which could provide a novel perspective for AD treatment [67].

#### In the CNS, the disordered TREM2-affected lipid metabolism contributes to the pathogenesis of AD

As discussed above, TREM2 was closely associated with the pathogenesis of AD and the lipid metabolism in the CNS. Considering that the disorder of lipid metabolism constitutes a major risk factor for AD, it would be interesting to figure out whether TREM2 affects the pathogenesis of AD by influencing lipid metabolism in the CNS.

Altered cholesterol metabolism related to TREM2 is associated with AD pathogenesis. R47H is a loss-of-function mutation of TREM2 and is related to an increased risk of AD [72]. Andreone et al. observed increased CE levels and myelin debris accumulation in TREM2(R47H) iMG compared to WT or TREM2 heterozygous iMG after exposure to myelin [43], which is consistent with the increase in CE levels in AD patients [73]. These results suggest that TREM2 loss-of-function might increase the risk of AD partly by suppressing normal TREM2-dependent cholesterol metabolism, although the specific mechanism remains unclear. The impaired uptake of Aβ complexed with cholesterol-rich LDL in Trem2 KO microglia might provide insights into future mechanistic studies [22].

Altered phospholipid metabolism also contributes to the pathogenesis of AD, possibly by interacting with AD pathologies such as Aβ and tau [74–78]. Interestingly, AD risk mutations of TREM2, including R47H, R62H, and H157Y, showed defects in binding to phospholipids, and the mutation with a larger risk of AD showed more significant defects [79]. Therefore, the increased incidence of AD related to these mutations may be partly due to the impaired binding of TREM2 to phospholipids [29]. Structural analysis of the binding of PS with TREM2 revealed the complex molecular mechanism of this phenomenon [52]. In addition, binding to phospholipids could induce the TREM2 intracellular signaling [29, 52]. Meanwhile, altered phospholipid metabolism may be a consequence of changed TREM2 function [26, 43]. These findings revealed an interaction between TREM2 signaling and phospholipid metabolism under AD conditions.

PLCγ2 is an intracellular enzyme that signals downstream of TREM2-DAP12. Recently, a PLCγ2 mutation (P522R) that results in a functional hypermorph that enhances the PIP2-metabolizing function of PLCγ2 has been identified as a protective mutation against AD [53, 65, 80]. Considering that PLCγ2 can be activated by stimulating the TREM2-DAP12 signaling pathway, this finding suggests that TREM2 might affect the pathogenesis of AD by influencing phospholipid metabolism.

In addition, TREM2 is indispensable for the transition of microglia to DAM. While there are both pro-inflammatory and anti-inflammatory phenotypes, the latter shows enhanced lipid metabolism and plays a protective role in AD. Additionally, as a major risk factor for AD, APOE might cooperate with TREM2 in the metabolism of cholesterol and myelin and in the microglial transition to DAM. The close relationship between TREM2 and APOE further supports the hypothesis that TREM2 affects the pathogenesis of AD by influencing lipid metabolism in the CNS.

### TREM2 affects lipid metabolism in the periphery

**Fig. 3:**
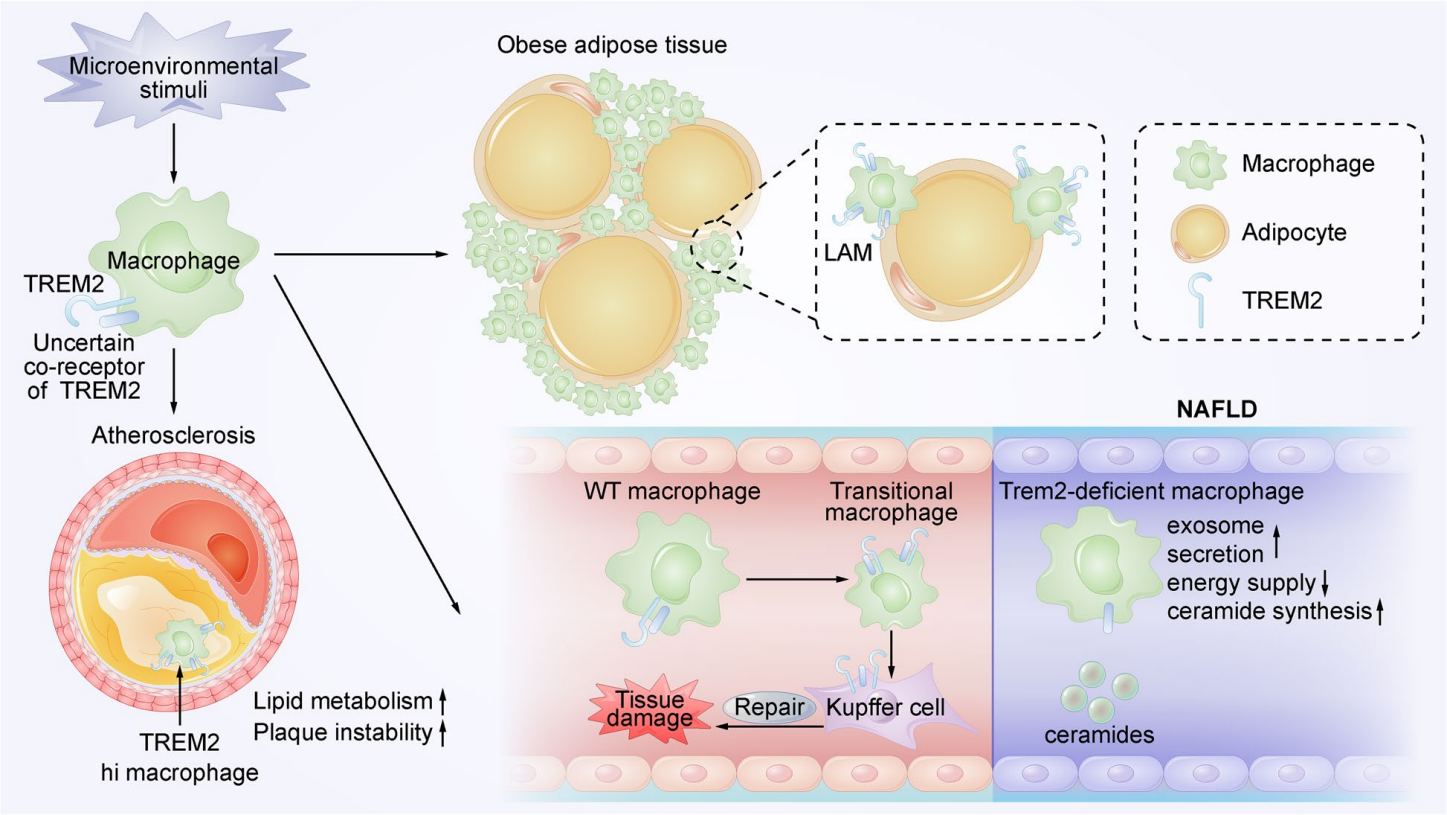
Macrophages with a high expression of TREM2 influence the metabolic comorbidities of AD. After sensing peripheral microenvironmental stimuli, macrophages transit into different phenotypes, such as LAMs, TREM2 hi macrophages, and hepatic transitional macrophages. LAMs play a protective role in obesity through the formation of crown-like structures in adipose tissue. TREM2 hi macrophages increase lipid metabolism in atherosclerotic plaques, although they may reduce the stability of plaques. The hepatic transitional macrophages express high levels of TREM2 and can then transform into Kupffer cells to promote the repair of liver tissue damage and influence the pathogenesis of NAFLD. In addition, Trem2-deficient macrophages in the liver increase the synthesis of ceramides and release exosomes that could impair hepatocytic energy supply in NAFLD

In addition to the CNS, recent studies have shown that lipoproteins and ApoE can also bind to and interact with TREM2 in the periphery [8, 15, 47, 79], suggesting that TREM2 might also be related to peripheral lipid metabolism. In fact, TREM2 can influence the development of obesity and its complications, such as hypercholesterolemia, atherosclerosis and NAFLD, in several ways, which will be discussed hereafter, including some lipid metabolism-associated mechanisms. Notably, these diseases show dysregulation of lipid metabolism, such as abnormal lipid accumulation, lipogenesis, and lipolysis [36–38]. They also alter the plasma levels of cholesterol, triglycerides and lipoproteins and change fatty acid (FA) metabolism pathways [37, 39–41], suggesting that the occurrence and development of these diseases further disrupt the homeostasis of peripheral lipid metabolism. Thus, the effect of TREM2 on obesity and its complications may be a possible mechanism by which TREM2 influences peripheral lipid metabolism. However, the accurate signaling pathways for TREM2 to influence lipid metabolism in the periphery, such as through TREM2-DAP12 or -DAP10 heterodimers, are not as clear as those in the CNS (Fig. 3).

Notably, diseases like obesity, atherosclerosis, and NAFLD are considered metabolic comorbidities of AD [32–35]. In addition, previous studies have found that high-fat diet (HFD), which is used to establish metabolic-associated disease models, disrupts the homeostasis of peripheral lipid metabolism, promotes the pathogenesis of AD, and upregulates the expression of Trem2 in AD mouse models [81, 82]. The disorder of peripheral lipid metabolism can induce peripheral inflammation and IR [83–86], which may lead to central inflammation and IR and further promote the pathogenesis of AD [87–90].

Therefore, in addition to discussing the effects and mechanisms of TREM2 on obesity and its complications in detail, we will also discuss the possibility that TREM2 influences the pathogenesis of AD by affecting the inflammation and IR induced by abnormal peripheral lipid metabolism.

#### The influence of TREM2 on obesity and hypercholesterolemia

The obesity epidemic has reached alarming proportions, with over two billion people worldwide suffering from overweight and obesity [91]. In addition, obesity is recognized as a central risk factor for several metabolic diseases, such as atherosclerosis, NAFLD, ischemic cardiovascular disease and type 2 diabetes [92, 93].

Recently, TREM2 has been linked to obesity. Previous studies have found that the expression of TREM2 is upregulated in the adipose tissue of obese animal models [94–96]. To further study the specific role of TREM2 in obesity, researchers conducted experiments on different types of Trem2 mouse models. By using transgenic (TG) mice overexpressing Trem2 both in the CNS and in the periphery, Park et al. found that TG mice had more adiposity and gained weight faster than WT mice when fed a HFD [94]. In addition, blocking Trem2 signaling restrained HFD-induced obesity in both WT and TG mice. Thus, these researchers proposed that Trem2 may promote diet-induced obesity by regulating the Wnt10b/b-catenin signaling pathway and adipogenic regulators [94]. Moreover, by comparing the results obtained from Trem2 TG mice and Trem2 blockade mice, these authors proposed that Trem2 may exacerbate adipocyte hypertrophy, inflammation, and IR during obesity.

However, another similar study conducted by Liu and his colleagues came to a different conclusion. They found that Trem2 − / − mice fed a HFD showed increased body weight, adipocyte hypertrophy, inflammation and IR, indicating that Trem2 plays a protective role in obesity [97]. It is not completely clear why these conflicting results occurred because there were no significant differences between the two experiments in diet and time point settings. In terms of diets, the main sources of fat in the HFD came from lard. The energy from fat differs from 45 to 60%; however, it is not plausible that this difference could cause a completely opposite result. Although the time they fed a HFD was different (from 12 to 16 weeks), the changes in body weight between the Trem2 KO/TG and WT groups occurred at the early stage and aligned with their final conclusions. It is likely that the Trem2 TG mice used by Park et al. are not suitable to study metabolic changes because Liu’s results are more consistent with current research on Trem2-driven lipid-associated macrophages (LAMs) [98].

LAMs are a subset of conserved TREM2 + macrophages that play a protective role in obesity mainly through the formation of crown-like structures in adipose tissue [98] (Fig. 3). By using obese mouse models, Jaitin et al. found that Trem2 deficiency inhibited the downstream molecular LAM program and eliminated the recruitment of macrophages to hypertrophic adipocytes. This leads to glucose intolerance, adipocyte hypertrophy, and body fat accumulation [98]. Combined with the lipid-sensing role of TREM2 in AD, these results indicate that Trem2 signaling is a primary pathway by which macrophages respond to the disorder of lipid metabolism.

Additionally, inactivation of the Trem2-related LAM program also led to hypercholesterolemia, which is a complication of obesity and an independent risk factor for AD. Considering the lipid homeostasis-maintaining role of LAMs, it is possible that Trem2 prevents hypercholesterolemia by maintaining lipid homeostasis in adipose tissue. However, it is not clear whether the protective effect of Trem2 against hypercholesterolemia is mediated by obesity.

However, recent bone marrow transplantation experiments found that hematopoietic/macrophage-expressed TREM2 reduces adipose hypertrophy in diet-induced obesity, but it exerts no influences on other obesity concomitant factors, such as IR and glucose intolerance. This study explained that IR and glucose intolerance in obesity were attenuated by TREM2 expressed on nonhematopoietic cells [99]. Although these researchers did not further identify the specific type of nonhematopoietic cells that protect against IR, it would be interesting to study the exact functions of TREM2 expression in adipose tissue during obesity.

In summary, TREM2 can influence obesity and its concomitant phenotypes, such as adipocyte hypertrophy, inflammation and IR. However, although the interactions among inflammation, insulin signaling and lipid metabolism have been widely discussed [100–103], it is unclear whether the effect of TREM2 on obesity is achieved by affecting these concomitant factors. Current evidence can only prove that TREM2 affects adipose tissue homeostasis under obese conditions. Although the specific role of TREM2 in obesity remains controversial, considering the given evidence, it is more likely that TREM2 serves as a protective factor that prevents the loss of lipid homeostasis. This is more aligned with its lipid metabolism-maintaining role in neurodegeneration as well as in hypercholesterolemia and other obesity complications described below.

#### The controversial role of TREM2 hi macrophages in atherosclerosis

Atherosclerosis is a lipid-driven inflammatory disease that is closely related to peripheral lipid metabolism [101] and is characterized by the formation of fatty plaques in great arteries. The plaques are rich in lipid-laden macrophages, which accumulate due to the recruitment of circulating monocytes as well as the differentiation and proliferation of local macrophages [104–106]. Recent studies have shown that macrophages are the most important type of immune cell in plaques in atherosclerosis [107, 108]. Generally, there are three main subtypes of macrophages, including resident-like macrophages, pro-inflammatory macrophages and anti-inflammatory TREM2 hi macrophages [109].

TREM2 hi macrophages are lipid-laden foamy macrophages that accumulate in the necrotic core of plaques. In addition, the levels of TREM2 expression in symptomatic human plaques were lower than those in asymptomatic human plaques [110], although the specific role of TREM2 hi macrophages in atherosclerotic plaques is still controversial. Recently, TREM2 hi macrophages have been identified as lipid modulators in plaques. Pathway analysis of TREM2 hi macrophages found enhanced regulation of lipid metabolism, such as cholesterol catabolism and efflux, revealing that TREM2 hi macrophages might have a protective role in foam cell formation and intracellular lipid accumulation [107]. However, the high expression of TREM2 may reduce the stability of plaques. In TREM2 hi macrophages, researchers observed increased levels of several types of cathepsins, which can enhance plaque vulnerability and inflammation in atherosclerosis [111, 112]. Recently, TREM2 hi macrophages were found to have nearly identical gene expression to osteoclasts and DAM [113]. Thus, TREM2 is thought to contribute to the calcification of atherosclerotic lesions [107], which might aggravate local tissue stress and damage plaque stability [114] (Fig. 3). In conclusion, additional mechanistic and functional studies are essential to investigate the specific function of TREM2 hi macrophages and explore its therapeutic potential in atherosclerosis.

#### TREM2 plays a protective role in NAFLD

NAFLD is the major cause of chronic liver disease [115] and is another complication of obesity [98, 116]. Interestingly, several studies have found that NAFLD is also a type of metabolic disease that can be regulated by TREM2. Trem2 deficiency in NAFLD-associated sepsis mouse models accelerate NAFLD progression [117], which is consistent with the observation that Trem2 − / − mice fed a HFD exhibited more severe hepatic steatosis [97]. A recent study further proved this finding and reported that Trem2 deficiency exacerbates hepatic steatosis in a fat and cholesterol level-independent manner. Moreover, restricting the increase in serum ceramides reversed adipocyte hypertrophy and hepatic steatosis in Trem2-deficient animals [99]. These findings suggest that Trem2 has a protective role against NAFLD progression, and the levels of serum ceramides might be a key part of this mechanism. Then, by using in vitro and in vivo models of liver lipid overload, Hou and his colleagues discovered that hepatic regulation of lipid overload involves partial Trem2-dependent metabolic coordination between hepatocytes and macrophages. They found that macrophages deficient in Trem2 could release exosomes, which impair mitochondrial structure and energy supply in hepatocytes. Additionally, Trem2-overexpressing macrophages increased the hepatic energy supply, revealing that Trem2-expressing liver macrophages may regulate hepatocytic energy metabolism during NAFLD [117]. Moreover, TREM2 contributes to the repair of chronic liver injury caused by NAFLD. A recent study identified a transitional subtype of macrophages that highly express TREM2 and is dominant during the recovery period of hepatocyte injury [118]. This phenotype of macrophages shows a TREM2-dependent transcriptional signature similar to those of DAM and LAM. They could then transform into resident-like macrophages (Kupffer cells), which express Trem2, to promote the repair of liver tissue damage [118] and influence the pathogenesis of NAFLD [119] (Fig. 3). The acquisition of transitional macrophages involves the upregulation of genes involved in oxidative stress responses and the downregulation of proinflammatory genes, which were not found in Trem2-deficient macrophages [118]. In summary, these studies describe TREM2 as a regulator of hepatocytic energy metabolism and liver lipid overload and as a promoter of macrophage phenotype conversion during liver tissue damage. Moreover, they further support the protective role of TREM2 in NAFLD.

Interestingly, the liver has been considered as a key organ involved in the development of AD as a result of its role in Aβ clearance [120]. Liver disease may lead to high levels of circulating Aβ due to its impaired detoxification ability [121]. This is consistent with the recent finding that synthesizing human Aβ only in the liver results in an AD-like neurodegenerative phenotype [122], which further confirms the pathogenic role of liver diseases, such as NAFLD, in AD [35].

#### TREM2-mediated inflammation and IR are potential pathways by which dysregulated peripheral lipid metabolism contributes to AD pathogenesis

In addition to the CNS, TREM2 in the periphery also plays a vital role in the development and progression of AD [17, 123]. Peripheral TREM2 mRNA levels are increased in AD and are related to AD-associated cognitive impairment and hippocampal atrophy, and the presence of the APOE ε4 allele further increases peripheral TREM2 expression [124]. We have discussed the influence of TREM2 on obesity, atherosclerosis and NAFLD, which are considered metabolic comorbidities of AD and disrupt the homeostasis of peripheral lipid metabolism. Notably, the disorder of peripheral lipid metabolism can induce peripheral inflammation and IR, which may lead to central inflammation and IR and further promote the pathogenesis of AD.

#### Inflammation

Dysregulated peripheral lipid metabolism can induce inflammation in the periphery., Macrophages can secrete inflammatory mediators and induce low-grade systemic inflammation upon the expansion of adipose tissue [125]. In addition, studies on mice with hyperlipidemia indicate that high circulating levels of lipoproteins [126], especially modified lipoproteins such as oxidized LDL [127], induce inflammation and promote the transition of anti-inflammatory macrophages into pro-inflammatory macrophages [83]. Fatty acid (FA) metabolism is a key part of the lipid metabolism system and is also closely associated with peripheral inflammation. Several studies have demonstrated that FAs regulate inflammatory pathways by activating Toll-like receptors (TLRs) [84] or binding to several G-protein-coupled receptors [128, 129] which are expressed on immune cells and metabolically active tissues. These findings are aligned with the observation that obesity [102] and its complications [56, 130] lead to peripheral or local inflammation. Interestingly, the inflammation present in these diseases is directly or indirectly affected by TREM2 [94, 97, 111, 112, 118], suggesting that TREM2 might influences inflammation induced by abnormal peripheral lipid metabolism.

TREM2 is known as a negative inflammatory regulator of macrophages [131] that antagonizes the response to proinflammatory stimuli by signaling pathways [131, 132] and enhances the expression of anti-inflammatory genes [30, 98]. Considering that excessive FAs induce inflammation by activating TLR [84] and that TREM2 can inhibit TLR signaling [132], it is possible that TREM2 plays an anti-inflammatory role upon dysregulation of lipid metabolism. Liu et al. found that macrophages in epididymal adipose tissue express more proinflammatory cytokines in Trem2 knockout mice than in WT control mice when fed a HFD [97], suggesting that Trem2 deficiency may upregulate the inflammatory response of macrophages. Similarly, Jaitin et al. found that Trem2-driven LAMs express many immunosuppression-related genes (such as LGALS1 and LGALS3), revealing that Trem2 is likely to restrain inflammatory response in adipose tissue [98]. A recent preprint found that TREM2 signaling inhibits the inflammatory response and promotes tissue remodeling in liver injury [118], and another study found that the frequency of LAMs was associated with the fibrosis score in the liver of nonalcoholic steatohepatitis patients [133]. These findings support the hypothesis that TREM2 may reduce pathologies in visceral adipose tissue by blocking inflammation.

However, whether TREM2 participates in the proinflammatory response in adipose tissues is still controversial. Park et al. conducted a study that had similar experimental conditions and methods as Liu’s study but used a transgenic mouse model overexpressing Trem2 [94]. These researchers found an increase in proinflammatory cytokines in the epididymal white adipose tissue of Trem2 TG mice compared with WT mice when fed a HFD [94]. These results are consistent with the findings of a recent single-cell RNA sequencing study [134], suggesting a proinflammatory role for Trem2. Nevertheless, in Park’s experiments, Trem2 promoted adipogenesis by upregulating peroxisome proliferator-activated receptor (PPAR)γ [94], an adipogenic regulator that could also inhibit inflammatory responses [135] and IR [102]. This TREM2-PPARγ pathway contradicts the role of TREM2 in promoting inflammation and IR, making these results confusing.

The inflammation induced by the dysregulation of peripheral lipid metabolism links obesity and its complications with AD [56, 89, 136]. Researchers have demonstrated that inflammatory challenges in the periphery trigger neuroinflammation [87], which can then contribute to the pathogenesis of AD in several ways, including interacting with Aβ metabolism [88, 137–139]. Considering that TREM2 is a widely discussed regulator of neuroinflammation [140], it is possible that TREM2 has a regulatory role throughout the dysregulated peripheral lipid metabolism-peripheral inflammation-neuroinflammation-AD axis.

#### Insulin resistance

IR is a disorder of glucose homeostasis, including decreased sensitivity to insulin in muscle tissue, adipose tissue, the liver, and other body tissues, regardless of elevated or normal glucose concentrations in blood [141]. IR is a major comorbidity of obesity [100], and the accumulation of FA metabolites in the liver and skeletal muscle and high levels of plasma free fatty acids (FFAs) may also lead to IR [85, 86]. These findings indicate an association between peripheral lipid metabolism and IR. Inflammation induced by dysregulated lipid metabolism is an important mediator of IR, and several studies have demonstrated that peripheral chronic inflammation leads to the impairment of insulin signaling and the consequent systemic IR [94, 142]. The activation of TLRs may be a mechanism underlying inflammation-induced IR. TLRs can be activated by increased levels of long-chain FAs in the obesity-related environment and thus contribute to the development of IR [143, 144]. Considering the inhibitory role of TREM2 on TLR signaling [132], it is possible that TREM2 might regulate IR induced by abnormal peripheral lipid metabolism.

Some studies have indicated that TREM2 can protect against IR. An insulin resistance test found that Trem2 − / − mice fed a HFD showed higher blood glucose levels than controls when challenged with insulin. Furthermore, an analysis of P-Akt protein levels in epididymal adipose tissue also indicated dampened insulin signaling upon Trem2 deficiency [97]. These results are aligned with a recent finding that the downregulation of TREM2 in adipose tissue is associated with advanced IR in patients with obesity [145]. Ceramides are important bioactive lipidic messengers that regulate cellular functions, including inflammation, survival and stress responses, and can induce IR during obesity and its complications [130, 146, 147]. Interestingly, a recent study used metabolomics to demonstrate an association between Trem2 deficiency and obesity-induced elevations in serum ceramides and found that Trem2 deficiency exacerbates diet-induced IR in a fat and cholesterol level-independent manner [99]. Inhibiting ceramide synthesis has been found to attenuate IR and to reverse adipose hypertrophy and secondary hepatic steatosis in Trem2-/- mice. These findings suggest that ceramide might be an important mediator by which TREM2 regulates IR [99]. In addition, the increased production of ceramide caused by hepatic steatosis promotes IR and can cross the BBB. Thus, it can lead to brain insulin resistance, neuroinflammation and neuronal apoptosis [148], indicating that ceramide may link IR and brain function.

Generally, IR can be divided into two categories, peripheral IR and brain IR. The latter is widely discussed but poorly defined. Peripheral IR is associated with dysregulated lipid metabolism, resulting in the overload of FFAs and the activation of cytokines in the periphery. Excessive levels of peripheral cytokines can cross and damage the BBB, leading to neuroinflammation and therefore inducing brain IR [89]. This mechanism has been supported by several experiments with rodent models that demonstrated that chronic peripheral IR may lead to brain IR as well as brain dysfunction [149, 150]. IR has been demonstrated to be an important risk factor for AD [90, 151, 152]. Brain IR leads to neuroinflammation, Aβ accumulation, tau phosphorylation, and brain bioenergetic dysregulation, possibly by regulating insulin-degrading enzyme and glycogen synthase kinase β [153, 154]. Thus, brain IR influences the pathogenesis of AD [102]. Most previous studies on TREM2 have focused on its influence on peripheral IR. However, there is a close relationship between peripheral and brain IR, and TREM2 regulates several pathogenic factors of IR in the CNS, such as abnormal inflammatory and immune responses [155]. Thus, we hypothesize that TREM2 may also have a direct or an indirect regulatory role in brain IR.

**Fig. 4:**
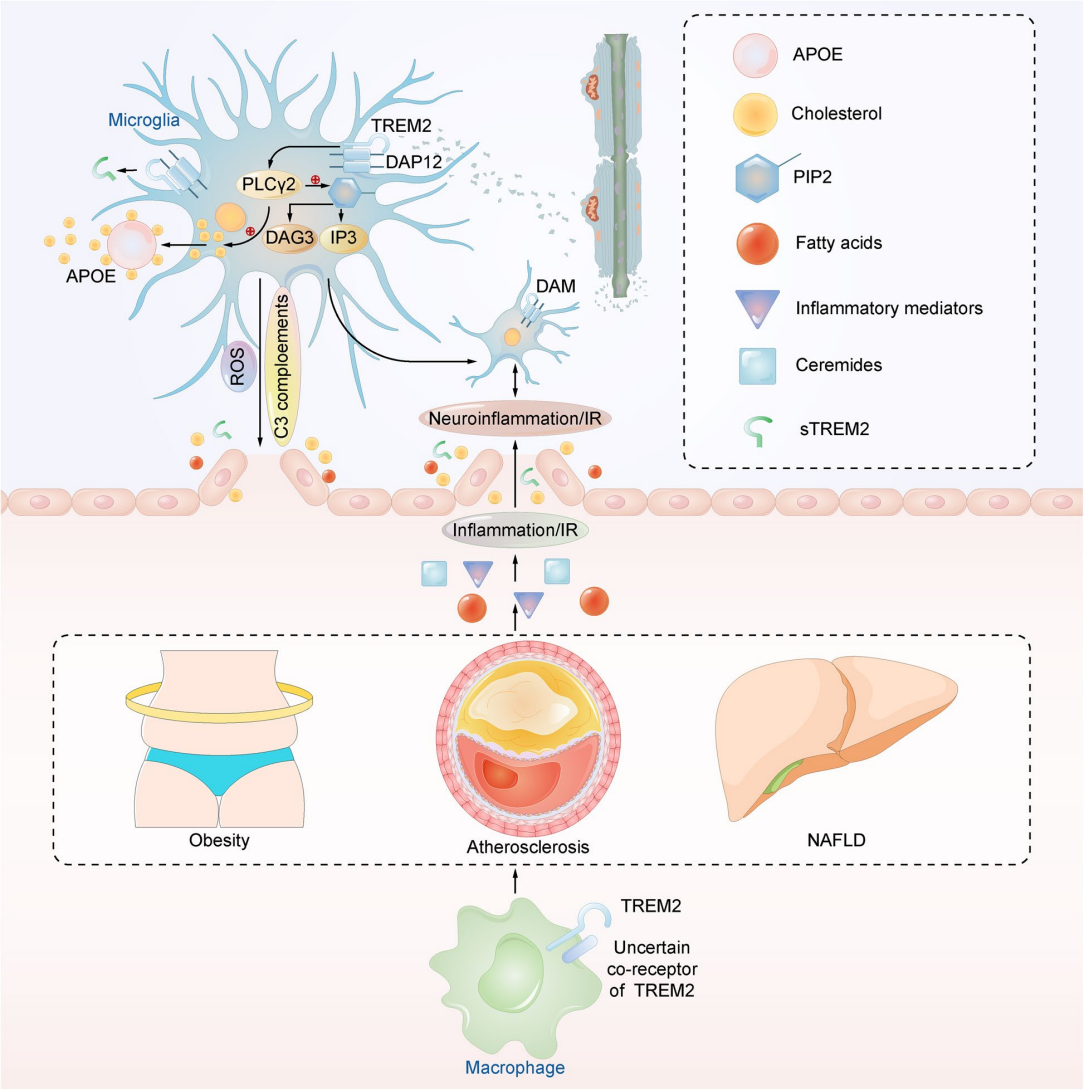
TREM2 is a regulator that links lipid metabolism in the CNS and the periphery. In the CNS, TREM2 promotes the microglial metabolism of cholesterol and PIP2 in a PLCγ2-associated way. TREM2 mediates the microglial response to myelin damage and promotes the microglial transition to DAM. In the periphery, TREM2-expressing macrophages can influence metabolic comorbidities of AD, such as obesity, atherosclerosis, and NAFLD. Therefore, the dysfunction of TREM2 leads to dysregulated lipid metabolism and induces inflammation and IR. Peripheral inflammation and IR eventually lead to neuroinflammation and IR in the CNS. In addition, the elevated sTREM2 levels in AD may cause BBB disruption. TREM2 may influence the integrity of the BBB by affecting inflammation, IR, the microglial oxidative response (releasing ROS), and C3 complement signaling. The damaged BBB allows for the increased passage of cholesterol and FFAs, therefore establishing an association between central and peripheral lipid metabolism

Collectively, these findings suggest that inflammation and IR are vital pathophysiological changes that can be induced by the dysregulation of peripheral lipid metabolism and are common pathways by which peripheral lipid metabolism influences the pathogenesis of AD. The majority of studies tend to support that TREM2 might maintain lipid homeostasis in the periphery and regulate peripheral inflammation and IR. This could subsequently influence the pathogenesis of AD (Fig. 4). However, Park et al. hold a different view that TREM2 might exacerbate obesity conditions and promote obesity-induced inflammation [94]. The reason for this contradiction is not completely clear, possibly because that the Trem2 TG mice used by Park et al. are not suitable to study metabolic changes. However, these results are similar to those that indicated that TREM2 promotes both the anti-inflammatory and pro-inflammatory phenotypes of DAM in the CNS [67]. As TREM2 is highly conserved throughout the body, it is also possible that the controversial role of TREM2 in regulating peripheral inflammation might be due to its similar dual effect on macrophage phenotypes.

In addition, the pathogenesis of AD is influenced by not only abnormal peripheral lipid metabolism but also TREM2-affected metabolic diseases, such as obesity [33], hypercholesterolemia [4, 156, 157], atherosclerosis [34, 56, 158] and NAFLD [136, 159]. The mechanisms by which these conditions influence AD pathogenesis are varied and do not act merely through the induction of inflammation and IR. Although the majority of current researches on the role of TREM2 in these diseases are in animal models, we hypothesize that TREM2 mutations might be genetic risk factors for the pathogenesis of these lipid metabolic diseases in humans. In the future, clinical studies could focus on exploring this point to establish a genetic association between peripheral diseases and AD.

### TREM2 may link lipid metabolism in the CNS and the periphery by influencing the integrity of BBB

Interestingly, the influence of TREM2 on central and peripheral lipid metabolism may not act through separate processes because apolipoproteins and lipoproteins can be regulated by TREM2 in both the CNS and periphery [47, 79, 160]. The BBB has an important role in linking the CNS and periphery, and the composition and function of the BBB are closely related to lipids, including phospholipids, cholesterol and sphingolipids [161]. Recent studies have found that soluble TREM2 (sTREM2) can cross the BBB and may disrupt the integrity of the BBB in the context of AD [162], possibly by interacting with pro-inflammatory proteins such as TNF receptor 1 and TNF receptor 2, and their effectors like intercellular adhesion molecule 1 and vascular cell adhesion molecule 1 [163]. The elevation of these molecules is considered to damage vascular endothelial function and the integrity of the BBB [164]. In addition, the BBB is vulnerable to disease conditions, including AD [165–167]. Cholesterol and FFAs have only a minimal ability to pass through the healthy BBB [168] but that their passage increases significantly when the BBB is damaged [169, 170]. Thus, we hypothesize that TREM2 may link lipid metabolism in the CNS and the periphery by influencing the integrity of the BBB (Fig. 4).

However, TREM2 does not have a confirmed effect on the BBB. Therefore, we summarized previous studies and proposed some potential mechanisms by which TREM2 may influence the BBB. The first mechanism acts by influencing inflammation and IR. Several studies have found that excessive inflammation in both the CNS and periphery can damage tight junctions and endothelial cells in the BBB and might exacerbate BBB impairment [171–173]. In addition, IR has been found to impair the tight junctions and pericyte coverage, with reduced expression of tight-junction proteins and transporters in the BBB [174, 175]. Given the previously described regulatory role of TREM2 in inflammation and IR under disease conditions, it is possible that TREM2 might influence the integrity of the BBB through these pathways.

The second potential mechanism by which TREM2 may influence the BBB acts through microglial oxidative stress. Several studies support the notion that microglial activation impairs the integrity of the BBB via reactive oxygen species (ROS) [176, 177]. Notably, recent findings have confirmed that the increase in TREM2 activity plays a critical role in regulating the activation of microglia [178, 179] and in generating oxidative stress [180]. RNA-seq analysis and semiquantitative RT–PCR studies have confirmed that the loss of Trem2 leads to a reduced microglial oxidative response in the brain [181].

Complement signaling is an acknowledged regulator of innate immunity in the brain, and it is the third potential mechanisms by which TREM2 may influence the BBB. Propson et al. found that enhanced complement (C3a/C3aR) signaling through endothelial cells could lead to dysfunction of the BBB [182]. Another study found that the expression of genes involved in the complement system, such as C4b, Cd18, and C3, were decreased in Trem2 KO mouse models compared with WT controls, suggesting that the loss of Trem2 may dampen the complement and innate immune systems in the brain [181]. Therefore, it would be valuable to figure out whether the effects of TREM2 on the brain complement system and the innate immune system can affect endothelial C3 signaling and thus affect BBB function.

Collectively, TREM2 may influence the integrity of the BBB by affecting inflammation, IR, microglial oxidative response and C3 complement signaling, and therefore it can link central and peripheral lipid metabolism. The influence of TREM2 on BBB integrity may be mediated by sTREM2 and the linking role of TREM2 allows researchers to consider lipid metabolism in the CNS and the periphery together to further investigate the relationship between lipid metabolism and AD.

### The potential and prospect of TREM2 as a therapeutic target for AD

Recent studies have started to focus on the therapeutic role of lipids in AD due to the involvement of lipids in BBB function, myelination, membrane remodeling, APP processing, energy balance, oxidation, receptor signaling, and inflammation. Regulating lipid metabolism is thought to be effective, and some studies have found that dietary supplements, including omega-3 fatty acids (DHA, EPA), and other types of lipid dietary modifications are likely to alleviate AD symptoms [183, 184]. In addition, using statins to alter cholesterol biosynthesis is considered to be a possible therapy [185, 186]. Treatment of an AD mouse model with atorvastatin was also found to regulate TLR4-mediated signaling and thus improve cognitive deficits. Considering that TLR4 expression could be upregulated by Trem2 deficiency [187], it is likely that statin treatment can ameliorate neuroinflammation more efficiently in patients with TREM2 mutations. Myelin-treated Trem2-deficient murine macrophages and human iPSC-derived microglia showed CE accumulation, which could be rescued by an inhibitor of acetyl-CoA acetyltransferase 1 [26]. This may therefore provide multiple beneficial effects on AD [188] and indicate that acetyl-CoA acetyltransferase 1 is an individualized therapeutic target for AD patients with TREM2 mutations. These findings suggest that therapies that reduce TREM2-associated lipid dysregulation in AD are likely to be feasible, which supports the value of the evaluation of TREM2 mutations in AD patients. Notably, although TREM2 mutations are present in only a small number of AD patients, the function and expression of TREM2 could be influenced by other genes involved in AD, such as TYROBP and APOE [124, 189]. These findings suggest that targeting TREM2-affected lipid metabolism for many patients with AD may be effective regardless of whether they carry a TREM2 mutation.

Considering that most of the AD-risk mutations of TREM2 are loss-of-function mutations, increasing the expression of TREM2 might be another therapeutic strategy for AD, even in patients without a TREM2 mutation. This may be achieved by using activating antibodies against TREM2 [8, 190] or ectopically expressing TREM2 in myeloid cells and then injecting them in vivo [191]. Price et al. have found that systemic administration of AL002a, an agonistic antibody of TREM2, can decrease Aβ deposition and improve cognition in 5XFAD mice [192]. Notably, a clinical trial on AL002 conducted by Alector and Abbvie (ClinicalTrials.gov NCT04592874) is now in the Phase 2 study. TREM2 expression is positively related to microglial uptake of Aβ-lipoprotein complexes and TREM2 deficiency reduced microglial uptake of Aβ complexed with ApoE-containing lipoproteins [22]. Interestingly, the influence of TREM2 deficiency is isoform-specific, as microglia expressing ApoE4 showed reduced uptake of Aβ that was additionally exacerbated by TREM2 deficiency compared with microglia expressing ApoE3 [62]. This finding further supports the therapeutic potential of TREM2 in patients carrying an APOE ε4 allele, which is the strongest genetic risk factor for late-onset AD. Furthermore, as mentioned above, TREM2 generally maintains lipid homeostasis in both the CNS and periphery. Thus, it is possible that enhanced expression of TREM2 alleviates dysregulated lipid metabolism during AD and AD metabolic comorbidities.

Recent studies have found that using adeno-associated virus or lentiviral particles to overexpress Trem2 alleviates Aβ deposition and cognitive deficits in APP/PS1 mice [193, 194]. In addition, bexarotene, a synthetic small molecule and specific retinoid X receptor (RXR) agonist, was found to have a therapeutic effect in AD mouse models. Bexarotene-treated APP/PS1 mice showed improved cognitive function, increased microglial phagocytosis and decreased Aβ plaque burden [195, 196], although these results remain controversial [197]. Genome-wide differential gene expression in the brains of AD model mice but not WT mice treated with bexarotene showed an upregulation of Trem2, Apoe, Tyrobp, and CD33, a microglial receptor that acts upstream of Trem2 [198]. Bexarotene affects AD by enhancing the immune response and inhibiting inflammatory reactions [199], which is similar to the function of the TREM2-TYROBP (DAP-12) signaling pathway in AD [200]. Thus, it is likely that the upregulation of Trem2, Tyrobp, and CD33 might mediate the therapeutic effect of bexarotene. In addition, treatment with bexarotene increases the lipidation of ApoE4 lipoprotein and improves cognitive function in AD model mice expressing human ApoE4 [201, 202]. Therefore, the upregulation of Trem2 caused by bexarotene treatment may increase the microglial uptake of Aβ complexed with ApoE-containing lipoproteins, which, as previously discussed, might be ApoE isoform-specific [62]. Future mechanistic studies are needed to confirm this interesting hypothesis.

However, it remains a challenge to locally increase TREM2 expression according to the clinical symptoms and laboratory tests of AD patients while avoiding the toxic effects of activating TREM2-expressing macrophages in other tissues, e.g., tumor growth and immune evasion. Drugs that upregulate TREM2 expression, such as bexarotene, showed side effects such as weight loss, hepatomegaly and difficulty breathing in mouse models [197, 203]. These results suggest that clinical treatments targeting TREM2 require further investigation.

## Conclusions

As mentioned above, TREM2 regulates lipid metabolism both in in the CNS and the periphery. In the CNS, TREM2 could regulate brain cholesterol and myelin metabolism and promote DAM activation in an APOE-associated manner. TREM2 signaling could be activated by binding with phospholipids and TREM2 may affect the metabolism of several types of phospholipids (Fig. 4). These alterations may influence AD by affecting AD pathologies or shifting the microglial response toward AD conditions. In the periphery, TREM2 can regulate lipid homeostasis and influence the inflammation and IR induced by dysregulated peripheral lipid metabolism. Thus, it might subsequently involve in the pathogenesis of AD, although the accurate function of TREM2 in this pathway is still controversial. However, although the effect of TREM2 on AD pathogenesis through lipid metabolism has been supported, more mechanistic and functional studies are needed to show how important lipid metabolism is in the TREM2-AD pathogenesis axis. Whether it is an indispensable factor throughout the axis or just an independent influencing pathway requires future exploration. In addition, we hypothesize that TREM2 may influence the integrity of the BBB by affecting inflammation, IR, microglial oxidative response and C3 complement signaling, and therefore it can link lipid metabolism in the CNS and the periphery. This linking role of TREM2 allows researchers to consider lipid metabolism in the CNS and the periphery together to further study the association between AD and lipid metabolism. Confirming the effect of TREM2 on the BBB is of greatest importance for verifying our hypothesis. Future studies should also attempt to explore the influence of TREM2 on BBB permeability and determine whether TREM2 molecules expressed in the CNS and the periphery can interact with each other.

Moreover, we discussed the role of lipids in TREM2-associated treatments for AD. Targeting the lipid dysregulation related to TREM2 dysfunction or upregulating TREM2 expression during AD are potential therapies worth exploring, although some significant problems remain to be solved.

## Data Availability

Not applicable.

## Abbreviations

AD: Alzheimer’s disease
APOE: Apolipoprotein E
Aβ: Amyloid-β peptide
BBB: Blood–brain barrier;
CE: Cholesterol esters;
CNS: Central nervous system
CPZ: Cuprizone
DAG: Diacylglycerol
DAM: Disease-associated microglia
DAP12: DNAX-activating protein of 12 kDa
DOK3: Downstream of kinase 3
ER: Endoplasmic reticulum
FAs: Fatty acids
FFAs: Free fatty acids
HFD: High fat diet
IP3: Inositol-1,4,5-trisphosphate
IR: Insulin resistance
LAM: Lipid-associated macrophage
LDL: Low-density lipoproteins
NAFLD: Nonalcoholic fatty liver disease
NAMPs: Neurodegeneration-associated molecular patterns
PE: Phosphatidyl ethanolamine
PI: Phosphatidylinositol
PI3K: Phosphoinositol 3-kinase
PIP2: Phosphatidylinositol 4,5-bisphosphate
PLCG2: Phospholipase Cγ2
PPAR: Peroxisome proliferator-activated receptors
PS: Phosphatidylserine
ROS: Reactive oxygen species
RXR: Retinoid X Receptor
TG: Transgenic
TLR: Toll-like receptors
TREM2: Triggering receptor expressed on myeloid cells 2
Tyrobp: Tyrosine protein tyrosine kinase binding protein
WT: Wild type
